# Abortion Lawfare and the Margin of Appreciation in the ECtHR Jurisprudence on Poland

**DOI:** 10.1007/s10691-025-09596-x

**Published:** 2026-03-13

**Authors:** Marta Bucholc, Iskra de Vries, Bartosz Cyran

**Affiliations:** 1 Faculty of Sociology, University of Warsaw, Karowa 18, 00-927 Warsaw, Poland; 2 Faculty of Social and Behavioural Sciences, University of Amsterdam, Amsterdam, the Netherlands; 3 Interdisciplinary Doctoral School, Faculty of Law and Administration, University of Warsaw, Warsaw, Poland

**Keywords:** Abortion, ECtHR, Poland, Margin of appreciation, Reproductive justice

## Abstract

This article examines the legal device known as the margin of appreciation at the European Court of Human Rights (ECtHR). To critically evaluate its modus operandi, we first explore scholarly literature on its functioning to then analyse the margin’s application in abortion-related jurisprudence. By relying on the framework of reproductive justice, we present three objections to the doctrine. First, spurious incrementalism addresses individualised burdens of standardised human rights procedures, which place disproportionate procedural and evidentiary constraints onto applicants and reinforce prevailing injustices. Second, politicised presentism explores the normative prioritisation of state representatives, which entrenches static abortion attitudes, even in the face of evolving societal views or the rights of minority groups. Third, the watershed of injustices interrogates the possibility of justifying restrictive jurisdictions through market logics, which displaces access to human rights across borders, compelling citizens to engage in a form of absurd forum-shopping for reproductive health care.

The inherent indeterminacy of human rights language gives rise to interpretive ambiguity, a phenomenon that becomes particularly pronounced in the context of abortion lawfare. Siri Gloppen defines abortion lawfare as the legal contestation over abortion rights, focusing on how legal tools and strategies are mobilised during political conflicts (2017; 2021). While advocacy organisations often mobilise human rights discourse to advance access to reproductive health care, the normative power of this framework ultimately remains in the hands of sovereign states (Baxi 2008).

This article examines the legal device known as the margin of appreciation to highlight how sovereign authority may sustain reproductive injustices and to better understand the iterative process of achieving European consensus on abortion (see also Peroni and Bucholc 2025). For the sake of readability, whenever we refer to the margin of appreciation, we use MoA, the margin, or the doctrine interchangeably. In essence, this doctrine preserves a degree of flexibility for national authorities in interpreting and applying the European Convention of Human Rights (ECHR), thereby accommodating the diversity of legal traditions and cultural contexts across member states. In highly sensitive matters, such as abortion, states are typically granted a wide margin, which allows for greater discretion in implementing the ECHR.

Our investigation takes place against the backdrop of numerous applications filed to the European Court of Human Rights (hereafter ECtHR or the Court) regarding access to reproductive health care in Poland. The parliamentary takeover by the political party Law and Justice (*Prawo i Sprawiedliwość*, PiS) in 2015 – marking a decline in the rule of law (Sadurski 2019) – fundamentally changed the legal landscape in Poland (see Krajewska 2021; Bucholc 2022a; Bień-Kacała and Drinóczi 2023). In October 2020, the discredited Constitutional Tribunal (K 1/20) banned nearly all access to abortion by eliminating severe foetal impairment as a ground for termination (see Council of Ministers 2021). This ‘judgement that wasn’t,’ as Aleksandra Gliszczyńska-Grabias and Wojciech Sadurski (2021) describe it – a ruling that bears the formal appearance of legality but lacks the substance of genuine judicial decision-making – was issued by a compromised, politically instrumentalised court that included unlawfully appointed judges. Nevertheless, the ruling entered into force, sparking widespread protests nationwide (Korolczuk 2016; Zabrzewska and Dubrow 2021).

During its eight years in power, PiS repeatedly violated European law^1^ and the ECHR^2^ due to its so-called ‘reform of the judiciary.’ However, the connection between abortion law and the rule of law emerged relatively late, only addressed by the ECtHR in *M.L. v Poland* in December 2023 (see Bucholc and Gospodarczyk 2024; Katsoni 2023; Nieróbca 2024; Zabrocka 2024).

Drawing on established ECtHR jurisprudence, this article argues that the margin of appreciation is susceptible to being used as an instrument of abortion lawfare, by mainly appealing to ethical, moral, and religious considerations embedded in, allegedly, national customs. To that end, we critically engage with the following body of ECtHR jurisprudence: *Tysiąc v. Poland* (2007), *A, B and C v. Ireland* (2010), *R.R. v. Poland* (2011), *P. and S. v. Poland* (2012), and *M.L. v. Poland* (2023).^3^ Our analysis of the Court’s case law mobilises the framework of reproductive justice, a feminist approach that emerged in the 1990s, building on the Black feminist tradition of the Combahee River Collective (Ross 2017). Unlike the narrow focus of the right to abortion, the reproductive justice framework foregrounds the positive obligations of the state to ensure equitable conditions that support individuals’ capacity to make reproductive choices and lead dignified lives (Chrisler 2013; Rebouché 2017; Ross 2017).^4^ This approach is grounded in critical race theory, which underscores the importance of examining the reciprocal construction of phenomena through categories of analysis (e.g., race, gender/sex, ability, or age) that, in turn, shape complex inequalities (Hill Collins 2015). For example, scholars have explored tensions between abortion legislation and disability justice (Francis 2023; Su and Lee 2025), as well as the dynamics of obstetric racism (Odems et al. 2024), and access to gynaecological care in rural areas (Plagens-Rotman et al. 2021).

So, unlike the pro-choice framework, which often reflects the experiences of more privileged individuals (Prince 2010, 43), reproductive justice offers a broader perspective, addressing not only access to abortion but also the mental, political and economic well-being of individuals and communities, as well as other related issues such as obstetric violence, ability to engage in family planning, and health care disparities. Relying on this framework, we argue that the circumstances reflected in selected ECtHR reproductive case law underscore the need for a consolidated European consensus and the destigmatisation of abortion. While some barriers to reproductive justice may be inherent to broader social and institutional contexts, the absence of such progress risks entrenching and exacerbating existing inequalities.

This article is structured around three central objections to the margin, each addressed in turn. To develop this critique, we begin by outlining the current state of the MoA. Next, we provide contextual background on the contemporary landscape of abortion law in Poland. Finally, we present the three objections to the margin’s current functioning: (a) spurious incrementalism, (b) politicised presentism, and (c) watershed of injustices.

## An Outline of the MoA and its Critique

The margin of appreciation, first articulated by the Court in 1976, is a context-sensitive legal device described as ‘a practice of negotiations’ (Přibáň 2017, 90). This tool allows signatory states discretion in interpreting and implementing human rights within their national constitutions, as it is designed to accommodate varying national moral and political sensitivities (see Shany 2005 for a detailed discussion). Importantly, the doctrine does not grant states carte blanche to infringe on human rights but provides a framework for balancing rights in situations lacking a clear consensus among member states.

The margin’s legal and political rationale is multifaceted. The Council of Europe (CoE) pragmatically justifies the doctrine by acknowledging the impossibility and undesirability of imposing uniform human rights standards across diverse national contexts. In this reasoning, states are seen as better equipped to assess moral and factual circumstances within their territories, thereby reducing potential confrontations between the Court and Contracting States over spheres of authority. Over time, several principles have been developed to guide the doctrine’s application, “with special emphasis on the principle of proportionality as a yardstick for evaluating whether the national authorities overstep the margin or not” (CoE, n.d.). The other principles include effective protection, subsidiarity and review, permissible interferences with Convention rights, and the European consensus standard (CoE, n.d.). Although a comprehensive exposition of these principles lies beyond the scope of this article, this chapter outlines how the margin operates as reflected in the Court’s jurisprudence, predominantly in view of reproductive matters.^5^

The pragmatic approach to the margin is reflected in the Court’s reasoning, which observes: “[t]here are different avenues for securing Convention rights, and even if the State has failed to apply one particular measure provided for by domestic law, it may still have fulfilled its positive obligation by other means.”^6^ Although the MoA has been criticised for a lack of conceptual specification, Jiři Přibáň explains how the doctrine’s ability to make the meaning of rights flexible contributes to the preservation of the ECHR’s general value (2017). It has also been argued that the doctrine plays a key role in protecting cultural particularities, especially in matters of religion, as highlighted by Dominic McGoldrick (2016), who underscores its function in preserving diversity. Importantly, McGoldrick also contends that human rights standards should evolve progressively, with the margin narrowing as ECtHR’s jurisprudence grows.

Nevertheless, reproductive cases frequently involve challenges in balancing the conflicting rights and interests of even higher complexity. In *Evans v. The United Kingdom*, the applicant sought protection of her right to have a genetically related child after her former partner had withdrawn consent to the storage of embryos created with the couple’s gametes.^7^ Their decision to enter IVF treatment was made shortly after the applicant had been diagnosed with ovarian cancer. Since the domestic law mandated that in the absence of one of the parties’ consent the embryos must be destroyed, the difficulty of the case stemmed equally from an irreconcilable “conflict between the Article 8 rights of two private individuals” and its entanglement in “a number of wider, public interests, in upholding the principle of the primacy of consent and promoting legal clarity and certainty” (§§73–74). The Court concluded that the state did not exceed the MoA afforded to it, due in part to the fact that the domestic regulation was adopted as “the culmination of an exceptionally detailed examination of the social, ethical and legal implications of developments in the field of human fertilisation and embryology, and the fruit of much reflection, consultation and debate” (§86).

Harmonising public interests and individual rights requires procedural safeguards for individuals whose rights are limited. The Court frequently refers to the principle of effective protection (effectiveness), as in the 2020 judgment in *Muhammad and Muhammad v. Romania*.^8^ Here, the Court emphasised that applying the margin cannot effectively nullify an individual right (§149). In this case, the lack of procedural measures to counterbalance the limitations imposed on the applicants meant the essence of their right was not preserved (§207). This procedural dimension of human rights violations was particularly significant in reproductive cases decided against Poland (*Tysiąc v. Poland* §117, 124; *R.R. v. Poland* §200, 208, 210; *P. and S. v. Poland* §§99–100, 108, 111). While each case addressed a different aspect of access to health care services, none concerned the right to abortion itself, as consistently noted by the Court (*Tysiąc v. Poland* §104; *R.R. v. Poland* §196; *P. and S. v. Poland* §89). Instead, the central issue was the state’s positive obligation under Article 8 of the Convention to establish minimal procedural safeguards for women seeking or considering an abortion in circumstances where abortion was (arguably) legal under national law (e.g., *R.R. v. Poland* §200).

Also central to the doctrine is the concept of consensus or “common ground” among member states of the CoE, which helps define the scope of a state’s discretion. In areas where there is no international or European standard, or where a common approach cannot be established, the states generally enjoy a wide degree of discretion. This reasoning permeates several ECtHR rulings on medically assisted reproduction, as well as those concerning the question of the beginning of human life, notably *Vo v. France* (2004).^9^ There, having observed “the diversity of views on the point at which life begins, of legal cultures and of national standards of protection” at the European level, the Court left the issue for the state’s discretion to determine (*Vo* §82). Thus, not only the means to reach compliance with Convention rights, but the very applicability and scope of the right to life under Article 2 fell within the state’s margin. The Court used a similar rationale to justify a wide margin of discretion with respect to regulating medically assisted reproduction, as it “gives rise to sensitive moral and ethical issues against a background of fast-moving medical and scientific developments, and since (…) there is no clear common ground amongst the member States” (*Evans* §81). The discretion accorded to the state extends to both the state’s decision to regulate this area, as well as to the content of such a regulation “in order to achieve a balance between the competing public and private interests” (§82).

As previously mentioned, the doctrine should narrow when there is evidence of legal consensus among states, as opposed to shifts in societal attitudes (Bamforth 2017; McGoldrick 2016). This principle is exemplified by *Fedotova and Others v. Russia*, where the ECtHR unanimously held that Russia had exceeded its margin by failing to provide legal recognition for same-sex couples.^10^ The Court’s reasoning relied on its own evolving case law concerning the rights of homosexual individuals (§§156–165), as well as a broader European trend toward recognising same-sex relationships (§§166–177). While the form and content of such recognition varied across member states, the Court identified “a clear ongoing trend” (§189). It reaffirmed that the Convention is a ‘living instrument’ requiring interpretation in light of contemporary conditions and evolving laws in democratic states (§§167–168). The principles reiterated in *Fedotova* were subsequently applied in *Przybyszewska and Others v. Poland* (2023), where the Court observed striking similarities between the arguments advanced by the Russian and Polish governments.^11^ Once again, the Court found that the state, in this case Poland, had overstepped its margin by failing to provide a legal framework recognising and protecting same-sex unions (§123).

Paradoxically, invoking European consensus can also expand a state’s discretion. This dual function becomes apparent when comparing *Fedotova* with *Vavřička and Others v. the Czech Republic* (2021), the latter concerning compulsory childhood vaccination and its repercussions for non-compliant parents.^12^ The Court assessed:

On the existence of a consensus, the Court discerns two aspects. Firstly, there is general consensus among the Contracting Parties, strongly supported by specialised international bodies, that vaccination is one of the most successful and cost-effective health interventions... Secondly, regarding the best means of protecting the interest at stake, the Court notes no consensus on a single model. Instead, there exists a spectrum of policies on childhood vaccination among the Contracting Parties... The Czech Republic has positioned itself at the more prescriptive end of that spectrum.(277-278)

Here, by balancing individual rights and public interests against the backdrop of the European consensus, the Court found the state’s limitations on the applicant’s right to private life proportionate to the legitimate aim of protecting children’s health (§Fedotova 306).

Clarifying the relationship between the MoA and the existence of a European consensus proves problematic (see also Nic Shuibhne and Niamh 2016), considering the judgement rendered in *S.H. and Others v. Austria* (2011).^13^ The applicants (two couples suffering from infertility) challenged the domestic legal ban on heterologous gamete donation, which for them was the sole possibility to have a child genetically related to one of the parents. Here, having first stated that it is not its task “to substitute itself for the competent national authorities” (§92), the Court seems to depart from its settled case law on the MoA. While concluding that “there is now a clear trend in the legislation of the Contracting States towards allowing gamete donation for IVF, which reflects an emerging European consensus” (§96), the ECtHR proceeded to downplay its significance:

That emerging consensus is not, however, based on settled and long-standing principles established in the law of the member States but rather reflects a stage of development within a particularly dynamic field of law and does not decisively narrow the margin of appreciation of the State (*d*).

Hence, it appears that in the highly sensitive domain of reproductive medicine, the Court remains hesitant to question the state’s authority, even in the face of a discernible legislative trend among CoE member states.

To conclude, the doctrine in its current form reflects a long tradition of jurisprudence encompassing diverse topics. This brief review of recent ECtHR case law highlights the doctrine’s pivotal role in resolving specific cases, including morally sensitive matters under Article 8 of the Convention, which is crucial to human rights jurisprudence on reproductive justice. The uncertain or ‘opaque’ results of its interplay with these principles raised criticism even among the judges in *S.H. and Others v. Austria*. Their joint dissenting opinion assessed that “through the combined effect of the European consensus and the margin of appreciation, the Court has chosen a minimum – or even minimalist – approach that is hardly likely to enlighten the national courts.”^14^ Scholars, too, have extensively analysed its applications and implications, expressing concerns about reinforcing the perception of international law as non-law. Debates about the doctrine’s utility frequently yield descriptive critiques – such as an ‘empty rhetorical device’ (Gerards 2018), ‘unpredictable’ (Greer 2017), ‘flawed in nature’ (Benvenisti 1999), and even ‘superfluous’ (Kratochvíl 2011) – urging the field to establish clearer, more testable criteria for its application. In response, several legal scholars have proposed re-conceptualisations or supplementary strategies to enhance its feasibility (Shany 2005; Kratochvíl 2011; Arnardóttir and Buyse 2016; Gerards 2018), while others have examined its logic in relation to specific national contexts, such as recent developments in Poland (Kapelańska-Pręgowska 2021).

### ECtHR and Abortion Law in Poland as of 2024

In Poland, following the collapse of authoritarian Communist rule in 1989, the public sphere underwent a process of “re-masculinisation” (Szelewa 2014), heavily influenced by the Catholic Church, which shaped the new official discourse and legal frameworks (Szelewa 2016). In 1993, the Act of Family Planning replaced the liberal abortion law that had been in effect since the 1950s,^15^ restricting access to abortion to three cases: threats to the woman’s life or health, severe foetal impairment, or pregnancies resulting from criminal acts. In all other instances, abortion was punishable by up to three years’ imprisonment (Article 152 §1 of the Criminal Code).^16^ Then, in January 1997,^17^ the socioeconomic basis for terminating a pregnancy was briefly reintroduced into Polish law but was deemed unconstitutional by the Constitutional Tribunal (K26/96) in May 1997 (see Kocemba 2025).^18^ Despite ongoing efforts to further restrict or liberalise abortion laws in Poland, the so-called ‘abortion compromise’ remained in effect until January 2021.

Poland faced troubling records of human rights violations in reproductive cases before the ECtHR. The three landmark cases – *Tysiąc* (2007), *R.R.* (2011), and *P. and S.* (2012) *v. Poland* – highlight this issue. In these judgments, the margin of appreciation was applied to balance the need to uphold domestic abortion laws with the state’s authority to determine their content (Kapelańska-Pręgowska 2021; Priaulx 2008). While these cases are widely cited in academic literature, their principles have not been implemented in Poland. Moreover,the ECtHR did not require changes to Polish abortion law in any of the cases but called for its consistent and reliable application. The failure to implement these judgments is perhaps best illustrated by numerous communications from Polish human rights NGOs, which frame these cases as symptomatic of systemic issues.^19^

The most recent change to the Polish abortion law involved tightening restrictions by eliminating the option to terminate pregnancies based on foetal defects.^20^ This October 2020 ruling by the Polish Constitutional Tribunal sparked a surge of legal challenges against Poland at the ECtHR. Agnieszka Kubal (2023) compellingly recounts the story of *Women’s Complaint*—a collective initiative comprising over one thousand applications submitted to the ECtHR by Polish women, supported by human rights lawyers and feminist activists. All the cases relate to women’s experiences in the wake of the tightened abortion restrictions. Although the ECtHR promptly notified Poland of several applications, the progress in these cases since then has been considerably less encouraging. This issue will be revisited in our first objection to the margin.

### Assessing MoA in Reproductive Cases: Three Objections

We argue that, in practice, the doctrine can enable states to reinforce oppressive and overly restrictive interpretations of abortion law. Our critique emerges from a long tradition of feminist thought that interrogates how legal structures reproduce hierarchical power relations (e.g., Brown 1992; Crenshaw 1989; Fraser 2013; MacKinnon 1989; Stuart Mill 1869). In doing so, we suggest that the doctrine can also be viewed as supporting a patriarchal and Eurocentric conception of state sovereignty, one that frames culture and tradition as monolithic, static, and border-bound (see also Henkin 1999; Cohen 2004; Pourmokhtari 2013). Such an understanding, in turn, obscures the internal diversity, contestation, and dynamism of cultural practices, ultimately legitimising state-centred narratives that serve dominant political interests rather than the lived realities of those most affected by restrictive reproductive regimes. To explain this, we present three objections to the current modus operandi of the margin of appreciation, which we situate within a broader strategic arena of abortion lawfare.

### Spurious Incrementalism: Small Steps Turning Infinitesimal

Building on the previously noted trend of a gradual narrowing in human rights interpretations within ECtHR jurisprudence, Janneke Gerards (2018) highlights how the margin of appreciation doctrine is increasingly being replaced by a strategic approach called incrementalism. This method entails a gradual, cautious, and case-sensitive process of developing new standards and criteria for human rights without provoking direct confrontation. Gerards (2018) illustrates this through *A, B and C v. Ireland,* demonstrating the slow evolution of criteria tied to respect for private life. Indeed, the selected cases reflect incremental developments in the application of Article 8 to issues of reproductive rights (*Tysiąc* §§107, 117, 119; *R.R.* §197; *A, B and C* §212). Yet, the Court’s handling of these cases remains highly dependent on the particularities of each complaint, its context, and the individual applicant involved. As a result, the gradual narrowing of the doctrine over time effectively shifts the burden onto applicants, making legal progress contingent on their personal resources and resilience—while also perpetuating the law-imposed reproductive labour disproportionately borne by women.

To discuss the margin’s incrementalism through the lens of reproductive justice, let us begin with one aspect of the Court’s approach: its requirement that applicants exhaust all domestic remedies (Article 35 §1 of the Convention) and independently provide evidence of their complaints (e.g., proof of consultations, written complaints). This requirement highlights systemic inequalities inherent in the human rights framework, dividing those with sufficient resources to meet strict filing conditions from those who lack them. In this sense, the requirement to exhaust domestic remedies does more than filter cases by procedural compliance; it also reproduces broader social hierarchies. While, in principle, such requirements privilege or disadvantage individuals based on any number of legally relevant capacities – such as financial resources, literacy, or access to legal aid – in the context of reproductive cases, they acquire a more specific function. Here, the procedural barriers interact with the state’s classification of citizens according to their perceived reproductive capacities and corresponding responsibilities, shaping who can effectively claim protection and whose claims are left unheard. As extensively discussed in feminist scholarship, such processes of classification are never neutral but instead reinforce existing hierarchies of, for example, gender relations (see e.g., Walby 1994; Yuval-Davis 1997). In the selected cases, the obstacles applicants face to reach the ECtHR are particularly severe. Complaints detail issues such as sham medical consultations, intrusive behaviour by medical staff, misleading information, psychological pressure, and manipulative tactics. As such, in both *R.R*. and *P. and S*. *v. Poland*, the Court found that the handling of the applicants’ cases by doctors and other persons amounted to the breach of Article 3.

In June 2023, the ECtHR dismissed eight complaints against Poland.^21^ The Court declared these cases inadmissible because none of the women provided reasonable or convincing evidence to substantiate their status as victims. The applications were deemed overly abstract and general, as the applicants failed to demonstrate that changes to domestic abortion law had infringed (or posed a risk of infringing) their rights (§§79–87). Another striking example concerns *D. v. Ireland*, where the case was declared inadmissible because the applicant had not exhausted domestic remedies, having failed to bring an action before the Irish courts (see also *P. and S.* §86).^22^ This decision starkly illustrates the administrative violence embedded in human rights commitments, where each case is treated in isolation from the broader, hostile reproductive socio-political climate.

The mandatory exhaustion of domestic remedies is a debilitating process that many cannot afford. To seek justice beyond one’s own state often requires enduring devastating and unbearable conditions. In the analysed cases, this burden has fallen overwhelmingly on the applicants, and we argue that it is precisely this burden that is central to understanding incrementalism: how many individuals must be dehumanised in documenting their distress before the ECtHR, and how much of that distress is reflected in the Court’s decisions?

Incremental progress in human rights protection comes through small steps, achieved at a disproportionate cost to applicants in reproductive matters. These steps remain limited because broader access to international human rights case law would require easing the applicant’s burden by challenging the scope of domestic legal remedies. States retain sovereign control over domestic legal procedures, which can only be contested when proportionality and effective protection are demonstrably violated. These principles, however, are tied to the margin and justified by considerations of cultural relativism. This is not about avoiding conflict or “stepping on toes”; those toes are shielded by the cast-iron boots of domestic procedural legislation, firmly defended by the doctrine.

Another factor limiting the pace of incremental progress in human rights protection is more apparent: the margin emphasises community traditions, history, and legal particularities, contrasting these with European consensus. In theory, this should lead to a narrower margin in cases where outliers to the consensus are few and clearly defined. In practice, however, the ECtHR’s ability to push for uniform human rights standards across CoE is constrained by the extent to which it defers to cultural and legal frameworks of domestic law, even when a European consensus exists. While the latter could theoretically justify narrowing the margin, there is no obligation to reduce it sufficiently to align outliers with the prevailing standard, as evidenced by the judgment in *S.H. and Others v. Austria* (§ 96, cited earlier). Further complicating matters, Poland’s EU Accession Treaty safeguards national sovereignty in morally significant areas, explicitly prohibiting EU regulation (Mishtal 2015). By acknowledging that abortion rights may be realised through various means while setting only non-realisation – ineffectiveness or extreme disproportionality – as the limit, the ECtHR fosters an illusion of rights protection. Instances of genuine contestation of state power remain rare. In cases of divergence, political opportunism and the prioritisation of state sovereignty invariably work against meaningful progress in human rights protections, further stalling incremental gains.

This is not to say that increments do not occur, but their promise far exceeds what the ECtHR is willing or able to deliver. The disproportionate burden placed on applicants yields results with minimal systemic impact, further diminished by delayed, selective, or non-existent implementation, as seen in the modest outcomes of reproductive cases against Poland.

In *The Most We Can Hope For…* Wendy Brown (2011) questions whether reducing individual suffering, as promised by the human rights framework, is the only utopia worth pursuing. In Poland, longstanding reluctance to promote sexual and reproductive autonomy, driven by fears of backlash from the Catholic Church, compounds with medical professionals’ hesitance or lack of expertise in providing legal reproductive health care (Mishtal 2015, 2019; Krajewska 2022) and the public-school system’s long-standing failure to deliver even minimal sexual education curricula (Davies 2020). The applicants’ experiences in the selected cases reveal the extensive discriminatory obstacles women face, requiring them to shoulder the burden of prolonged suffering to achieve compensation. In addition, we should remember that “international human rights are not designed as a form of collective power or vehicle of popular governance” (Brown 2011, 144). This underscores how small steps aimed at alleviating individual suffering become infinitesimal, incapable of driving bottom-up structural change.

### Politicised Presentism: Favouring Majoritarian Representation

By granting a wide margin of appreciation, the ECtHR links individual rights to the general interests of the community, safeguarding this balance through the principles of proportionality and effective protection. However, even when a community discriminates against a particular group by restricting their rights and access to public services, such actions may still be justified in the name of the general interest – grounded in national history, tradition, and culture – even if they diverge significantly from the European consensus. This tension between community justifications and broader European standards underscores the need for a socio-political critique of legal objections to spurious incrementalism. Such a critique must assess and incorporate the lived experiences of those most affected by restrictive abortion laws, ensuring equitable access to reproductive health care free from discrimination or undue state interference.

Adopting a more critical stance than the CoE’s definition of the doctrine, Benvenisti’s (1999) core concern comes into focus: whether the political majority is both willing and able to protect the interests and safety of minorities? From the outset, it is evident that the MoA is not designed to prioritise those on the social periphery. Even *private life*, as protected under Article 8 – drafted as a “broad term” (§107 *Tysiąc*) and its boundaries “not clear cut” (§112 *Tysiąc*) – is framed in alignment with the interests of the community as a whole (§111 *Tysiąc*). In addition, the Court leaves the definition of “community” vague and open to interpretation, whereas states are granted deference under their domestic constitutions to determine whether a fair balance has been struck between individual rights and the public interest (§214).

The ambiguity between individual rights and public interest appears to have been strategically leveraged by the applicants in *A, B and C v. Ireland*, where the changing public views on abortion have been explicitly used to challenge the restrictive abortion law and advocate for greater recognition of reproductive rights. By highlighting shifting societal attitudes, arguing that “the will of the Irish people has changed since the 1983 referendum” (§223), the applicants sought to demonstrate that the balance between individual autonomy and public interest must evolve accordingly, pushing the Court to reconsider traditional deference to national authorities in the context of abortion. Crucially, the Court recognised a growing European consensus favouring access to abortion on broader grounds than those permitted under Irish law (§235). In doing so, it once again sidestepped the contentious issue of defining when human life begins (see *Vo v. France*, §85), instead grounding its reasoning in the more flexible international consensus on abortion law (*A, B and C,* §§235, 237).

The applicants in *A, B and C v. Ireland* strategically emphasised the evolving nature of collective morals from a bottom-up perspective. They argued that, although the restrictions they faced might have been “in accordance with the law,” this interpretation reflected the Government’s view—not their own (§169). This highlights how the applicants challenged the state’s foundational, sovereignty-oriented approach (Follesdal 2016) and rejected what could be termed politicised presentism: the tendency to conflate a society’s moral consensus with its current legislative framework and government at a specific moment in time.

Despite these efforts – and citing the state’s allegedly superior position in such matters (§§223–226) – the Court deemed the opinion polls supporting the applicants’ claims insufficient (§226). For example, the Court referenced the Maastricht Treaty of 1992, which prohibited interference with Article 40.3.3 of the Irish Constitution. This reasoning reflects the ECtHR’s deference to sovereignty, subsidiarity, and national democracy. However, by acting in that manner, the Court risks abandoning its critical role as an external safeguard against potential state tyranny. As Eva Brems (2023, 902) observes, a wide margin can lead to “interpretational flexibility” for contracting states, allowing them to “minimise their own responsibility for human rights protection” (see also Iglesias Vila 2023).

Political presentism embedded in the doctrine tends to absolutise the political will of elected rulers, which may contradict or disregard the beliefs of significant segments of the population (see also Lee 2021) . After all, only 21 per cent of the Polish population believes that abortion should be prohibited in cases where it is known that the foetus has a severe disability (CBOS 2023). It is therefore reasonable to conclude that the current law does not reflect this. In abortion lawfare terminology, this quality of the margin further fuels “state lawfare,” which hints at a particular use of legalistic means for political gain (Goppen 2017, 6). Elsewhere, Marta Bucholc and Iskra de Vries (2025) illustrate how the issue of abortion in Poland has been simultaneously mobilised as a political weapon and deployed as a strategic gambit in struggles over political power.

Thus, political presentism effectively erases dissenting voices, as governments are accepted a priori as the legitimate and representative agents of their societies. In the context of Poland, this dynamic is a central feature of abortion lawfare: the strategic deployment of legal mechanisms to contest or entrench abortion regulation. Law, here, is a site of ongoing struggle. In such cases, national sovereignty is invoked not to reflect consensus, but to obscure internal contestation and suppress marginalised perspectives. This underscores the need to scrutinise who speaks for “the public” in legal settings, and whose voices are excluded from the construction of the law.

An alternative approach to the MoA must not only recognise the empirical margins of society that the doctrine fails to represent, and who are therefore excluded from meaningful access to human rights, but also address the structural conditions that produce this exclusion. It must adopt an empirical perspective on societal dynamics to counter politicised presentism. Follesdal (2016) likewise critiques the normative prioritisation of sovereignty and proposes a “person-centred” version of the margin, which would prioritise human rights protection, including state violence against its citizens.

The doctrine’s emphasis on recognising and accommodating cultural differences can, on the surface, be seen as something to celebrate. However, politicised presentism highlights the law’s power in shaping and regulating its subjects. The top-down application of the margin, aligned with the government in power, perpetuates exclusionary practices that enforce culturally determined performative citizenship (Isin 2017). This, in turn, reinforces the idea of the sovereign state as the ultimate guarantor of rights (Butler 1990, 3). The safeguarding of the so-called “Polish traditions” is thus not merely a judicial issue; it also sustains imposed and essentialising narratives about what Poland purportedly represents. As such, we argue that politicised presentism serves as a legal instrument to justify and perpetuate structural injustices, enabling these systemic inequalities to persist.

To tie up loose ends, sovereignty as an absolute legal principle appears to shield the normative values represented by the state. In Poland 2015–2023, these values reflected the unilateral right-populist agenda of the ruling authorities (Gwiazda 2021). The notion that only developments initiated by the legislature can represent society – despite the much slower pace of legal change compared to shifts in societal attitudes – effectively stifles bottom-up political opposition. Domestic dissent and contestation are pushed into the margins, relegated to amicus briefs. The growing importance of reproductive justice over recent decades, driven by societal ruptures and developments, underscores the urgency of recognising such changes as dismissing these signals only reinforces the sovereign status quo.

Considering ongoing knowledge production, globalisation, and the growing diversification and hybridity of cultural and religious identities within states, the classical concept of sovereignty increasingly appears as anachronistic. Nancy Fraser’s theory of post-Westphalian democratic justice similarly highlights the limitations of a justice framework that centres on *what* justice entails while insufficiently interrogating for *whom* it is intended. As Fraser (2013, 257) argues: “Today, many question the territorial state as an appropriate unit of thinking about justice. The effect is to destabilise the previous structure of political claims-making – and therefore to change the way we argue about social justice.”

### Watershed of Injustices: Individualised Forum-Shopping of Human Rights

Our final objection to the doctrine challenges the overly simplistic separation of intrastate and interstate relations. The ECtHR’s very foundation as a human rights court rests on this distinction, which is increasingly difficult to justify within the social reality of the CoE as a multicentre legal system (see Łętowska 2010). The CoE comprises closely interconnected societies, with more than half of its member states also part of the EU, whose fundamental freedoms foster even greater connectivity and interdependence.

The core issue of the watershed of injustices stems from a certain market logic, justifying restrictive laws by pointing towards the possibilities of obtaining certain matters beyond state borders. As a result, individuals are effectively pushed into a form of absurd forum-shopping for human rights—forced to seek protection in other jurisdictions. Rather than being upheld by the state, the burden of securing fundamental rights is displaced onto individuals, who must navigate complex legal systems and selectively pursue rights across borders. This phenomenon is particularly significant in the context of reproductive justice. For example, in *S.H. and Others v. Austria* (§114):

“the Court also observes that there is no prohibition under Austrian law on going abroad to seek treatment of infertility that uses artificial procreation techniques not allowed in Austria and that in the event of a successful treatment the Civil Code contains clear rules on paternity and maternity that respect the wishes of the parents”

(see also *A, B and C*§239).

In *A, B and C v. Ireland* (§241), the Court concluded that, since Irish citizens had the legal right to travel abroad for abortions and access information and medical care in Ireland, “the prohibition in Ireland of abortion for health and well-being reasons, based as it is on the profound moral views of the Irish people as to the nature of life,” did not exceed the discretionary power granted to the Irish state. The Court determined that Ireland had struck a “fair balance” between the applicants’ rights and those invoked on behalf of the unborn. However, the notion of fairness employed by the Court resembles that found in competitive sports: anyone who knows the rules has a fair chance of winning, regardless of inherent inequalities between players.

Out-of-state travel for abortion – institutionalised as a systemic mechanism in Irish law during the period examined in *A, B and C v. Ireland* – has become a pan-European phenomenon. Despite its scale, research on abortion-related travel across borders remains limited. Notably, the ERC-funded BAR2LEGAB project traced the trajectories of women seeking legal abortions in selected Central and Western European countries (De Zordo et al. 2020; Zanini et al. 2021). The study revealed that such travel, primarily driven by gestational age limits (less frequently by other restrictions), involves thousands of women annually moving from Western European countries with stricter abortion laws to those with more liberal regimes. Indeed, these patterns provide strong indirect evidence that transborder abortion-related mobility would be significantly higher in cases where women seek abortions entirely prohibited in their home countries. For such cases, only rough estimates are available. Moreover, research consistently shows that travelling for reproductive health care, including abortion, is costly, logistically challenging, and often distressing if not traumatising.

In fact, in *A, B and C v. Ireland*, the Court acknowledged that “travelling abroad for an abortion constituted a significant psychological burden on each applicant” (§126) and described it as “both psychologically and physically arduous” as well as “financially burdensome” (§163). Similarly, in *M.L. v. Poland,* the applicant was forced to travel abroad for an abortion after the planned legal procedure was cancelled following the 2020 Constitutional Tribunal ruling. The applicant alleged violations of her rights to private and family life, freedom from inhuman or degrading treatment, and the right to a fair trial, arguing that the restriction was not “prescribed by law” (§§73, 124) due to the Tribunal’s lack of impartiality and improper composition. Indeed, the Court found that the Tribunal’s interference with the applicant’s rights was unlawful and that the experience of an out-of-state abortion “caused her pain and suffering and had a significant psychological impact on her” (§84, 101). However, the Court concluded that the requirement to seek an abortion abroad did not, in itself, amount to a violation of human rights, Article 3 in particular (§84, 85). To further elaborate on *M.L v. Poland,* the MoA was repeatedly invoked by the *amici curiae* supporting the legality of abortion restrictions and the refusal to perform the procedure by medical staff, as well as by the dissenting opinion of judges Paczolay and Wojtyczek. Interestingly, the ECtHR avoided addressing the margin in its judgment, instead focusing on the lawfulness of the interference with the applicant’s rights. The interference was deemed unlawful because it resulted from a Tribunal judgment that lacked legal authority to infringe on individual human rights. Thus, the case was resolved without assessing whether such interference would have been lawful had the Constitutional Tribunal been a bona fide court. Notably, the dissenting opinion (§2) argued that the doctrine should influence not only the scope of interference with human rights but also its lawfulness.

Considering the burdens of out-of-state abortion, one might expect the principles of effective protection and proportionality to argue against increasing intrastate disparities in human rights protections that systematically and persistently produce such outcomes. Ironically, this very situation bolsters the application of the doctrine as a reinforcement of state sovereignty. The ECtHR appears to rely on a curious double fiction. On the one hand, it upholds state sovereignty, the political representation of citizens, and the primacy of national legislation as reflecting societal morals and worldviews. On the other hand, fully aware of the interconnectedness of CoE societies, it applies the margin freely, knowing the diversity of CoE states and population mobility may provide remedies, while leaving the golden calf of state sovereignty untouched.

Feminist scholarship has often criticised the ECtHR for its handling of abortion travel cases (see Katsoni 2023). However, the direct connection between abortion travel and the Court’s doctrine is rarely addressed. Only in a system of geographically close-knit and culturally, economically, and logistically interconnected states can sovereignty and control over citizens be maintained by using cross-border health travel as a buffer to safeguard human rights. Access to this buffer, however, is unevenly distributed. The right or ability to travel illustrates this disparity: while some women can obtain reproductive care with relative ease, others must overcome substantial legal, financial, and logistical barriers simply to obtain the same protections (see O’Shaughnessy 2024).

The watershed principle is most clearly illustrated by abortion travel cases but applies broadly to other scenarios where human rights recognised in some CoE states are denied in others, forcing individuals to travel or relocate to exercise these rights. Examples include same-sex marriage, recognition of parenthood, and adoption for same-sex couples, as seen in the communicated cases of *A.D.-K. and Others v. Poland,*^23^ and *A.P. and R.P. v. Poland*.^24^ In such instances, the burden of securing justice shifts directly onto individuals, with states serving merely as intermediaries—limited to actions such as permitting travel (explicitly addressed by the ECtHR in *A, B and C*) and providing information to citizens. Each instance of such injustice undermines the effectiveness and proportionality of human rights protection. States evade their responsibility to establish equal standards, instead delegating this effort to individuals with minimal institutional involvement. Ultimately, these injustices hinder the development of a coherent European consensus. By granting a broad margin of appreciation to outlier states, the ECtHR effectively legitimises such disparities, enabling governments to sideline CoE human rights standards while upholding discriminatory laws to the detriment of their citizens.

## Conclusion: How Much Consensus is Enough?

This contribution has argued that the current modus operandi of the margin of appreciation may enable its appropriation as a form of abortion lawfare (Gloppen 2017; 2021), embedding Eurocentric nationalist politics and patriarchal power structures within the international human rights framework and legitimising their continued influence. By presenting three objections to the margin’s functioning – (a) spurious incrementalism, (b) politicised presentism, and (c) watershed of injustices – we suggest that the degree of discretion afforded to states can operate as a strategic legal tool though which governments can manipulate procedural requirements, moral narratives, and institutional authority to suppress dissent and restrict access to reproductive health care (see also Berms 2023). This dynamic is particularly evident in the realm of reproductive justice, where the ECtHR’s deference to state sovereignty has allowed restrictive regimes to persist under the guise of cultural legitimacy and democratic representation.

As an international judicial body, the ECtHR’s cautious approach to decision-making is tactically understandable, especially in cases involving abortion laws in states that diverge significantly from the European consensus. But is abortion truly an exceptional or outlier issue? As Julia Kapelańska-Pręgowska aptly noted:

*A, B and C v. Ireland* was decided by the Grand Chamber of the ECtHR in 2010. Have the abortion laws in Council of Europe member states undergone any changes since then? Currently, 41 out of 47 [46 as of 2024, after Russia’s leaving the CoE in 2022] Council of Europe countries allow abortion on request or on broad social grounds (i.e., well-being). The most restrictive abortion laws remain in force in Andorra, Malta, and San Marino. Liechtenstein and Poland (since 2021) allow abortion only when a woman’s life or health is at risk or when the pregnancy is the result of sexual assault. In Monaco, a third ground is permitted: a severe foetal anomaly. It means that the European consensus has become consolidated, and Poland is the only country where abortion has been restricted. The key question is whether this even stronger consensus will be regarded as significant and capable of narrowing the margin of appreciation (2021, 219).

This last question resonates strongly with our argument that addressing the issue at its core requires a fundamental reconceptualisation, and, consequently, a narrowing of the margin.

By affording a wide margin of discretion, the ECtHR reduces its incremental steps to an almost negligible scale, rendering the process of gradual change in its jurisprudence largely superficial. The doctrine’s prioritisation of state sovereignty further entrenches politicised presentism in the ECtHR’s approach to state representation. State-made laws, viewed as reflections of the prevailing normative orders and moral beliefs within societies, are privileged over alternative perspectives. In societies with deeply divided opinions, such as abortion, this approach reinforces political presentism—directly contradicting the Court’s mission to protect the rights of individuals and minorities.

Political presentism and spurious incrementalism create a double bind for groups challenging dominant views in the name of human rights. From a reproductive justice perspective, the doctrine functions as a structural barrier within ECtHR doctrine, reinforcing cultural determinism and exclusion through its reliance on effectiveness and proportionality. Within this logic, watersheds of injustices emerge—particularly when restrictive abortion laws are justified by pointing to alternative jurisdictions, perpetuating exclusion and prioritising state sovereignty over reproductive justice, disproportionately affecting marginalised and economically vulnerable women.

Paradoxically, a mechanism intended to accommodate diversity instead enforces cultural homogeneity, fostering conflict rather than genuine pluralism. This type of lawfare for cultural determinism is often framed in contemporary EU relations as a form of ‘new colonial dependence’ (Bucholc 2022b, 43; see also Korolczuk and Graff 2018). While ostensibly respecting plural values and the semantic variation of human rights across societies (David 2020), the margin instead facilitates national exceptionalism that undermines genuine diversity. This is particularly pertinent in Poland, where abortion law remains constrained by the 2020 Constitutional Tribunal ruling, despite the Tribunal’s judgment being deemed an unlawful interference with individual rights in *M.L v. Poland* and attracting widespread criticism.

Although a more doctrinally grounded decision might have offered a stronger critique of Poland’s approach, hundreds of abortion-related applications, some communicated to the Polish government as early as 2021, demonstrate the ongoing conflict between national restrictions and CoE human rights protections.^25^ It seems unlikely that all pending cases will be decided solely based on the unlawfulness of interference, underscoring the critical role of the margin and the urgent need for its feminist critique.

The future application of the margin will be decisive not only for Poland but also for states, as it will define whether the ECtHR serves as a genuine guardian of reproductive justice or as a tool that entrenches state control and systemic exclusion. Its decisions will shape not only the legal landscape in Poland but the broader trajectory of human rights across Europe. In this moment, the Court’s approach is not merely procedural—it is decisive, moral, and transformative. The stakes could not be higher.

## Footnotes

1 Most recently in Case C-204/21 (Disciplinary Chamber); earlier cases include judgements in C-487/19 (nomination to the Chamber of Extraordinary Control and Public Affairs), C-791/19 (new disciplinary regime in the judiciary), C-824/18 (nomination of judges to the Supreme Court), C-585/18 (independence of the Disciplinary Chamber of the Supreme Court), C-192/18 (independence of ordinary courts) and C-619/18 (independence of the Supreme Court).

2 Xero Flor w Polsce sp. z o.o. v. Poland (app. no. 4907/18), Broda and Bojara v. Poland (app. no. 26691/18 and 27367/18), Reczkowicz v. Poland (app. no. 43447/19), Dolińska-Ficek and Ozimek v. Poland (app. nos. 49868/19 and 57511/19), Advance Pharma sp. z o.o. v. Poland (app. no. 1469/20), Grzęda v. Poland (app. no. 43572/18), Żurek v. Poland (app. no. 39650/18), Wałęsa v. Poland (app. no. 50849/21).

3 Tysiąc v. Poland, App. no. 5410/03, Judgement of 20 March 2007; A, B and C v. Ireland, App. no. 25579/05, Judgement of 16 December 2010; R.R. v. Poland, App. no. 27617/04, Judgement of 26 May 2011; P. and S. v. Poland, App. no. 57375/08, Judgement of 30 October 2012; M.L. v. Poland, App. no. 40119/21, Judgement of 14 December 2023.

4 Although this article primarily uses the term “women” for clarity and consistency, we acknowledge that reproductive experiences are not limited to women. Our use of “women” is not intended to exclude these groups but reflects prevailing terminology in the relevant ECtHR case law.

5 The variety of the Court’s interpretative and argumentative tools is analysed in Mężykowska and Młynarska-Sobaczewska (2023).

6 *H.F. and Others v. France*, App. nos. 24384/19 and 44234/20, Judgement of 14 September 2022, para 252.

7 *Evans v. The United Kingdom*, App. no 6339/05, Judgement of 10 April 2007.

8 *Muhammad and Muhammad v. Romania,* App. no. 80982/12, Judgement of 15 October 2020.

9 *Vo v. France*, App. no. 53924/00, Judgement of 8 July 2004.

10 *Fedotova and Others v. Russia*, App. nos. 40792/10, 30538/14 and 43439/14, Judgement of 17 January 2023.

11 *Przybyszewska and Others v. Poland*, App. nos. 11454/17 and 9 others, Judgement of 12 December 2023, para 116.

12 *Vavřička and Others v. The Czech Republic,* App. no. 47621/13 and 5 others, Judgement of 8 April 2021.

13 *S.H. and Others v. Austria*, App. no. 57813/00, Judgement of 3 November 2011.

14 See the joint dissenting opinion of Judges Tulkens, Hirvelä, Lazarova Trajkovska and Tsotsoria to the majority conclusions in *S.H. and Others v. Austria*.

15 The Act of 7 January 1993 on Family Planning, Protection of Human Foetus, and Grounds for Permitting the Termination of Pregnancy (Dz. U. 1993, No. 17, item 78), Polish text online: https://isap.sejm.gov.pl/isap.nsf/DocDetails.xsp?id=wdu19930170078. Accessed 6 August 2025.

16 The Act of 6 June 1997, The Criminal Code (Dz. U. 1997, No. 88, item 553), Polish text online: https://isap.sejm.gov.pl/isap.nsf/DocDetails.xsp?id=wdu19970880553. Accessed 6 August 2025.

17 The Act of 30 August 1996, amending the Act on Family Planning, Protection of Human Foetus, and Grounds for Permitting the Termination of Pregnancy as well as other legal statutes (Dz. U. 1996 No. 139, item. 646), Polish text online: https://isap.sejm.gov.pl/isap.nsf/download.xsp/WDU19961390646/O/D19960646.pdf. Accessed 6 August 2025.

18 Constitutional Court of Poland, Judgment of 28 May 1997 (Case no. K 26/96), Polish text online: https://ipo.trybunal.gov.pl/ipo/Sprawa?cid=6&sprawa=310. Accessed 6 August 2025.

19 See e.g. the Communication from Helsinki Foundation for Human Rights (5 November 2021), available in the ECtHR ‘Case documentation’ online: https://hudoc.exec.coe.int/#%7B%22execidentifier%22:%5B%22DH-DD(2021)1337revE%22%5D%7D. Accessed 6 August 2025.

20 Constitutional Court of Poland, Judgment of 22 October 2020 (Case no. K 1/20), Polish text online: https://isap.sejm.gov.pl/isap.nsf/download.xsp/WDU20210000175/O/D20210175.pdf. Accessed 6 August 2025.

21 See Decision of 8 June 2023 in *A.M. against Poland* and 7 other applications, available online: https://hudoc.echr.coe.int/eng#%7B%22itemid%22:%5B%22001-225355%22%5D%7D. Accessed 6 August 2025.

22 Decision of 28 June 2006 declaring inadmissibility of *D. v. Ireland*, App. no. 26499/02.

23 *A.D.-K. and Others v. Poland,* App. no. 30806/15, communicated on 26 February 2019.

24 *A.P. and R.P. v. Poland,* App. no 1298/19, communicated on 8 December 2020.

25 Notification of 12 applications concerning abortion rights in Poland (applications nos. 1819/21, 3682/21, 4957/21, 6217/21, 3639/21, 4188/21, 5876/21, 6030/21, 3801/21, 4218/21, 5114/21 and 5390/21), 8 July 2021, available online: https://hudoc.echr.coe.int/fre-press#%7B%22itemid%22:%5B%22003-7074470-9562874%22%5D%7D. Accessed 6 August 2025.
